# Case Report: Virtual Reality Neurofeedback Therapy as a Novel Modality for Sustained Analgesia in Centralized Pain Syndromes

**DOI:** 10.3389/fpsyt.2021.660105

**Published:** 2021-04-20

**Authors:** Nnamdi Orakpo, Ulrick Vieux, Cristian Castro-Nuñez

**Affiliations:** ^1^Department of Psychiatry, Garnet Health Medical Center, Middletown, NY, United States; ^2^Transitional Year Program, Garnet Health Medical Center, Middletown, NY, United States

**Keywords:** virtual reality, neurofeedback, analgesia, centralized pain, neuromodulation

## Abstract

Neurofeedback (NFB) Therapy is a form of biofeedback, using the electroencephalogram (EEG) that has been in use since the 1970s, serving as a non-pharmacological intervention for epilepsy and psychiatric conditions such as anxiety, depression, insomnia, PTSD, post-concussive syndrome, and now, centralized pain. Chronic pain can increase neuronal activity and eventually causes poor modulation of pain messages. With the emergence of Virtual Reality (VR) in acute pain management, and the contraindications of opioids in chronic pain, applying novel biotechnologies seems like the next frontier in multimodal pain management. In this study, the VR and NFB technologies were fused together (VR-NFB) and used as a novel treatment modality for a 55-year-old woman who suffered from chronic pain secondary to spondylolisthesis with cervical, thoracic, and lumbar disc herniations after a motor vehicle accident with comorbid depression, anxiety, sleep deprivation, and difficulty with activities of daily living, and inability to participate in physical therapy. Our case reports on the sustained analgesia achieved for 1 year after a trial of VR-NFB, and the usefulness of neuromodulation in centralized pain syndromes.

## Introduction

In neuropsychiatry, it is known that 20–40 NFB sessions are required for the neurofeedback's rehabilitating effect to be permanent. Because of neuroplasticity, the brain has the capacity to store traumatic experiences as well as learn new, healthy behaviors through new synaptic connections. This intervention was designed in the 1970s to activate the brain's inherent self-repairing mechanisms. Traditional neurofeedback has been shown to influence modulation of pain messages reaching the primary pain processing centers of the brain. The theory of central sensitization syndrome indicates that chronic physical pain becomes cognitive and emotional pain—or brain pain. Prolonged bombardment of the dorsal horn of the spinal cord possibly stimulates increased activation of neurons *via* the anterior spinothalamic pathway (ascending), leading to sensitization of the brain (1, 2). Chronic pain messages are modulated by the anterior cingulate gyrus, dorsolateral prefrontal cortex (DLPFC), and the insular cortex, as the three major postsynaptic pain-processing centers. Additionally, the thalamus and somatosensory cortex are involved in modulating pain messages (3), as well as the amygdala, under the direction of the medial prefrontal cortex (MPFC), which is involved in catastrophizing pain (4), and the affective component of the pain experience. Glutamate and substance P are two of the many neurotransmitters that are involved in lowering the threshold for the firing of the unmyelinated C fibers (5).

Virtual reality (VR) is a novel technology that has been shown in various studies to reduce acute pain as well as serving as an immersive distraction to pain. This process is probably carried out by the prefrontal cortex (PFC), which is responsible for the blocking of negative thoughts and feelings, distractions, and emotional regulation (6). In 1965, Melzack and Wall (7) came up with the gate theory that suggests that the amount of focus on pain, the emotional response to the pain, and the painful experience collectively influence how the brain will perceive the pain (7). Based on Wickens' (8) theory on multiple resources, the resources in different sensory areas of the brain all operate autonomously. It is obvious that the VR technology integrates the visual, auditory, and tactile sensory functions, while simultaneously distracting patients from their pain. Hoffman et al. (9) found that immersive VR during physical therapy helped lower pain scores significantly for burn victims. Another study by Sarig-Bahat et al. (10), found that a single session of VR alone where the patient sprayed flies in the game, improved range of motion in the cervical spine and reduced neck pain. Decades of research on NFB therapy has demonstrated the usefulness of this tool in mood disorders, sleep disorders, ADHD, neurological rehabilitation and, more recently, chronic pain. Research has shown that VR alone can reduce acute pain scores and minimize a patient's focus on pain. In this study, both technologies were combined as a novel clinical tool (VR-NFB) and was evaluated for its effectiveness as a treatment modality and its sustained analgesia for centralized pain.

## Case Description

**Figure 1:**
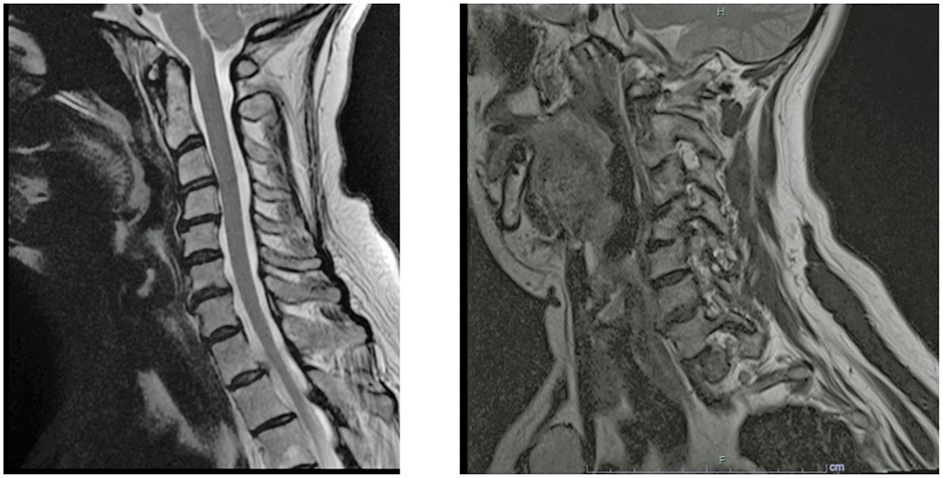
MRI impression: Bilateral foraminal stenosis at C4 and C5, related to uncovertebral arthropathy, with exiting C5 nerve root impingement. At C5–C6, there is significant right foraminal stenosis with exiting C6 nerve root impingement. Photo courtesy of Joseph A. Marchione, MD, Neuroradiologist at Garnet Health Medical Center.

A 55-year-old woman with a past medical history of cervical spine stenosis with radiculopathy, post-concussive syndrome, sciatica, status post motor vehicle accident (MVA), with a past psychiatric history of depression, anxiety, chronic pain syndrome, and PTSD related to the MVA, who presented with persistent right-sided shoulder and neck pain. She reported poor sleep quality and stated that she was not yet a candidate for surgery per the four different orthopedic and neurosurgical evaluations she underwent. She was visibly anxious, irritable, and tearful as she replayed the MVA in her mind. “Every time I come to a stop sign, my palms sweat, my heart races, I tense up, paranoid that someone will hit me from behind each time.” She had recently visited the emergency room for acute exacerbation of her cervical pain with complaints of stiffness, right upper extremity radiculopathy, worsening neck pain, decreased range of motion (Figure 1), and inability to sleep and perform activities of daily living (ADLs) (Figure 1). She was prescribed the following oral medications: meloxicam 15 mg, gabapentin 100 mg three times daily, amitriptyline 10 mg nightly, cyclobenzaprine 10 mg as needed and memantine 5 mg per day. She reported that gabapentin had partially helped in the past but was no longer effective.

## Imaging

### MRI of Cervical Spine

#### Treatment and Clinical Course

Materials and software used are the Oculus Rift-S VR headset (2019), the EEG NeuroAmp, and the Cygnet Software for the neurofeedback functions, all provided by BeeMedic. The patient was assisted with placing the headset over her head and over the leads for maximum comfort, and she was given prompts to stare at the game she wanted to play so it could load and begin. The patient underwent a clinical trial of 20 sessions of VR-NFB Therapy, completing two sessions weekly for a total of 10 weeks. The patient was screened with a thorough clinical history prior to starting VR-NFB. She had no history of thalamic strokes or traumatic brain injury, which were exclusion criteria. The patient was optimized at a frequency of 0.15 mHz at T3–T4, and T4–P4 at 0.175 mHz, for 20 total sessions. The Wong-Baker Pain Scale was used to identify the level of pain she experienced on a daily basis, as well as the level of anxiety, sleep, depression, PTSD symptoms, fatigue, and any interference in IADL/ADLs associated with the pain. Before each session began, she was asked about her level of distress using Subjective Unit of Distress before (BSUD) and then again at the end (ESUD) to observe for any acute change in the metrics.

Generally, the patient continued to struggle with fluctuations in pain intensity and reported an average pain score of 6 prior to starting VR-NFB sessions, with an average pain score of 4.5 status post VR-NFB session (15% improvement per session).

**Figure 2:**
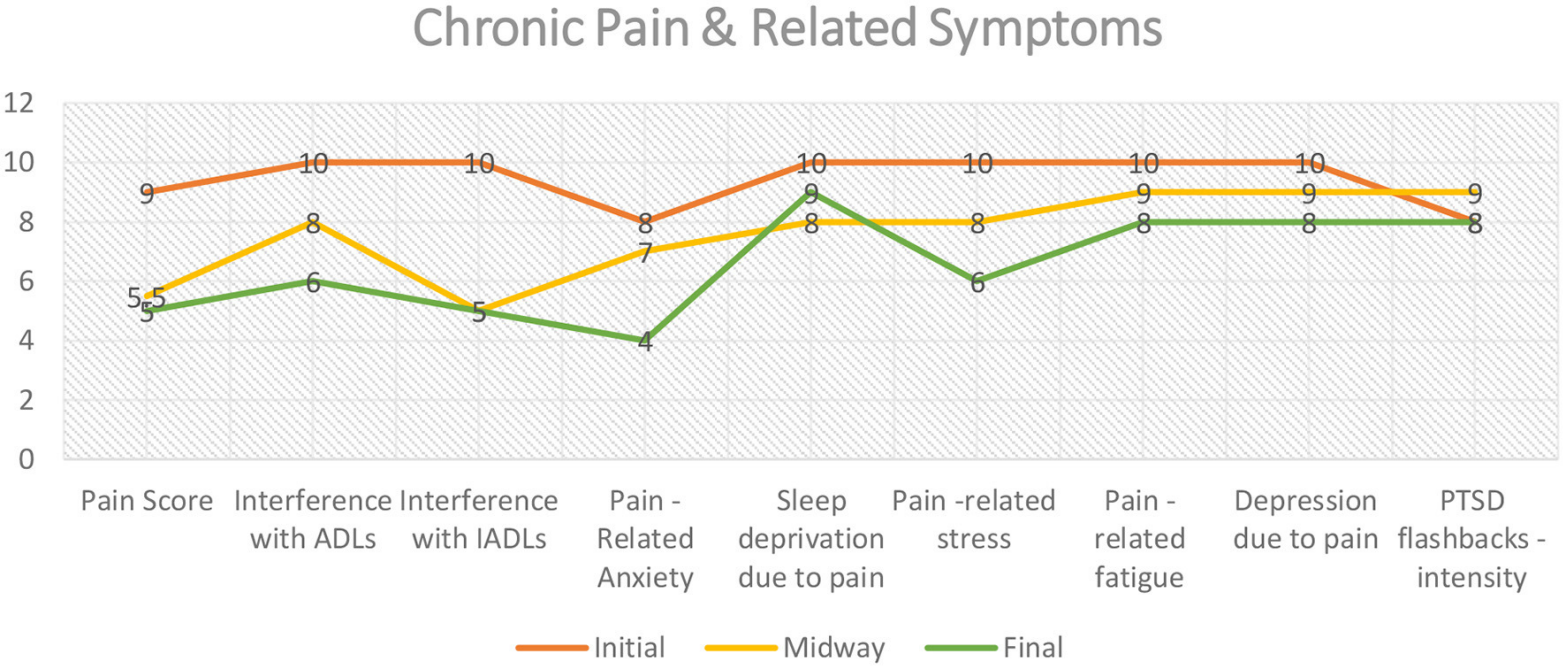
Chronic pain intensity & pain-related symptoms.

After 20 VR-NFB sessions the patient was evaluated for any change to her symptoms since starting the clinical trial. Her pain score (see Figure 2) improved from 9 to 5, indicating a 40% decrease in pain at the end of the trial. Her activities of daily living (ADLs) improved by 40% while her independent activities of daily living (IADLs) improved by 50%. Patient initially reported pain-related anxiety with a score of 8, which decreased to 4 after 20 VR-NFB sessions (40% improvement). She initially reported trouble falling asleep and staying asleep because of her chronic pain, reporting sleep deprivation as 10 out of 10, improving only to an 8 (20% improvement) after the clinical trial. Her scores for pain-related fatigue and pain-related depression were similar to that of sleep deprivation with a 20% improvement. She had no change in the intensity of her PTSD flashbacks post therapy vs. initial (Figure 2). As for her medications, she was able to completely discontinue gabapentin for neuropathic pain and cyclobenzaprine for cervicalgia and neck spasms; she began taking meloxicam less frequently because “it practically does nothing anymore.” She continued her amitriptyline at the same dose and frequency.

## Post-Treatment Follow Up

**Figure 3:**
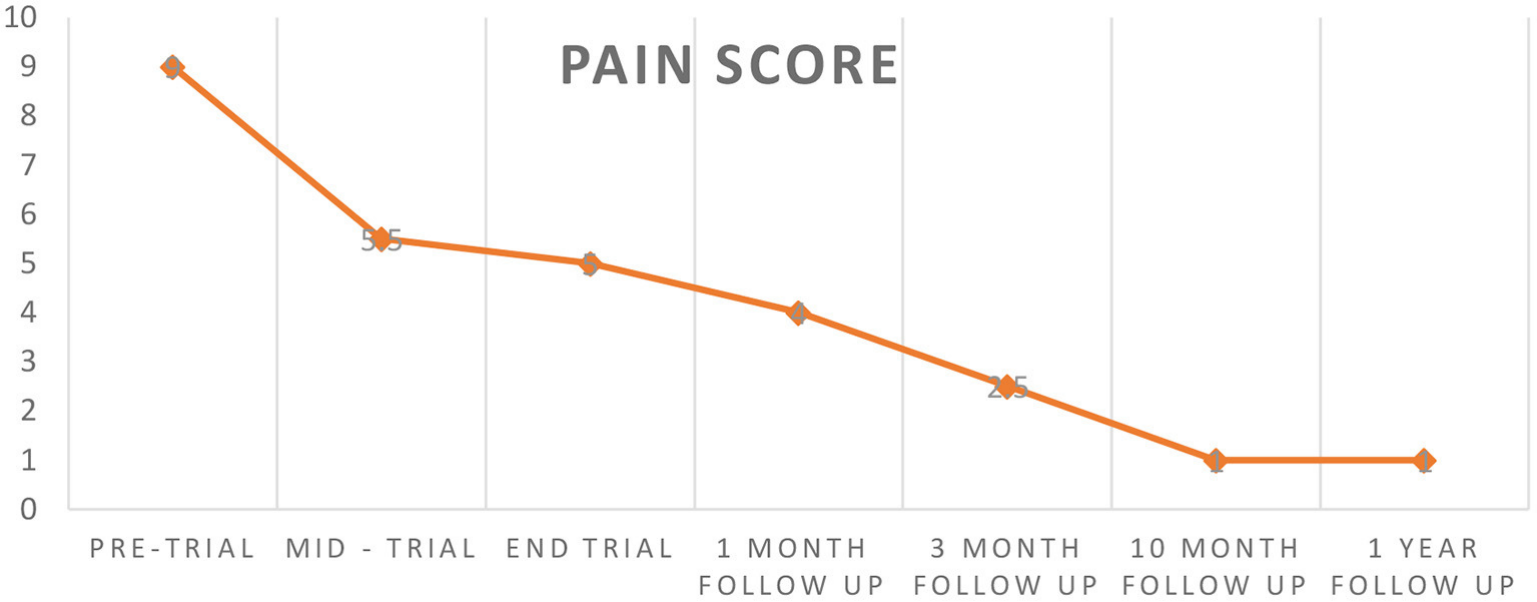
Pain score—pre, mid, end trial and follow up—please refer to text.

At the end of the 20-session trial, she reported that her pain was at 5 out of 10 (40% improvement). The research team followed up with the patient at 1, 3, and 10 months. At 1 month, she reported that her pain score was at 4 out of 10, and declining. At the 3 months follow up, she reported that her pain continued to improve, reporting a pain score of 2.5 (mild) out of 10, and she reported a pain score of 1 at the 10-month and 1-year post trial follow up, indicating an overall improvement in pain of 80% from initial to 1-year follow up (Figure 3). This improvement in pain has allowed her to participate in physical therapy, while maintaining analgesia.

## Discussion of Outcomes

This study explores VR-NFB as a non-opioid treatment for chronic pain. Virtual Reality Neurofeedback (VR-NFB) helped the patient experience an 80% improvement in pain from initial to 1 year follow up, with improvements in ADLs, anxiety, depressive symptoms, and sleep, while discontinuing Gabapentin for neuropathy, muscle relaxants, high dose Naproxen, and TCA medications known for cardiac side effects. In fact, literature and clinical data are reporting the role of pain, particularly chronic pain, as an important stressor on mental health and its relationship with everyday world stimuli. At her 1-year follow up, the patient reported that analgesia was sustained, her pain was more manageable, and she stated that the pain is hardly noticeable. She reported feeling happier, less anxious, and she was sleeping more than 6 hours nightly. She stated that her colleagues noticed she was brighter and less irritable, and less depressed to the extent of being able to discontinue amitriptyline with the help of her primary care physician.

## Limitations and Strengths

The fact that this is a case report is an obvious limitation, and the study could possibly be improved by using other measurements for the affective components of pain like the Patient Health Questionnaire (PHQ-9) for evaluating the severity of depression and response to therapy, or the Beck Anxiety Inventory (BAI), used to measure severity of anxiety weekly. The strengths of this study is its focus on alleviating chronic pain from a neuropsychiatric perspective, and its employment of novel technologies to mitigate opioid dependence and reduce polypharmacy and improve resilience. Further, a strength of this study was that the VF-NFB therapy helped the patient achieve sustained analgesia for more than 1 year compared to the short-term pain relief in previous studies (9, 10) that used VR alone.

## Conclusion

While traditional forms of neurofeedback have been shown to improve acute and central neuropathic pain in other studies, and VR alone has been shown to improve acute pain, VR-NFB is a novel approach that incorporates an additional analgesic effect of immersive distraction in the short term. This additional analgesia may be related to a beta endorphin-induced increase in dopamine in the mesolimbic pathway of the brain when a patient is engaged, experiencing pleasure, excited, seeking reward, or exploring newness. This acute increase in dopamine may enhance compliance in chronic pain patients, as they find pleasure in the VR-NFB therapy and its rewards, while actually anticipating therapy. Through its modulatory effect and change in neurochemistry, this novel treatment proved effective in sustained analgesia, and may provide permanent pain relief. We will follow up with the patient in 2 years to determine if the effects are permanent. Our case reports a 55-year-old woman who was able to experience long lasting pain relief and discontinue multiple medications that have cardiac side effects. This novel treatment could assist psychiatrists and practitioners in psychology in augmenting cognitive behavioral therapy for chronic pain (CBT-CP), while inadvertently eliminating polypharmacy. VR-NFB may serve as an adjunctive therapy in the toolkit of multimodal pain management. We have provided evidence that this therapy is non-invasive and effective in sustaining pain relief, while improving ADLs, anxiety, depression, and sleep, and eliminating polypharmacy, leading to overall improved resilience and self-regulation.

## Data Availability Statement

The original contributions presented in the study are included in the article/supplementary material, further inquiries can be directed to the corresponding author.

## Ethics Statement

The studies involving human participants were reviewed and approved by Dr. Cleveland Lewis, MD, Dr. Pamela Murphy MD, and Dr. Eleonora Feketeova. The patients/participants provided their written informed consent to participate in this study.

## Author Contributions

NO initiated the concept, design, data collection, analysis, and interpretation of the study. UV contributed to the final revision of the manuscript. CC-N contributed to the revision of the final manuscript. All authors contributed to the article and approved the submitted version.

## Conflict of Interest

The authors declare that the research was conducted in the absence of any commercial or financial relationships that could be construed as a potential conflict of interest.
